# Sleep quality of adult psychiatric outpatients at Chris Hani Baragwanath Academic Hospital

**DOI:** 10.4102/sajpsychiatry.v29i0.2113

**Published:** 2023-11-29

**Authors:** Celeste M. Harlies, Wendy Friedlander

**Affiliations:** 1 Department of Psychiatry, Faculty of Health Sciences, University of the Witwatersrand, Johannesburg, South Africa

**Keywords:** psychiatric disorders, psychiatric outpatients, sleep quality, sleep disorders, insomnia, Pittsburgh Sleep Quality Index, PSQI

## Abstract

**Background:**

Sleep disorders are increasingly prevalent among the general population and individuals with mental disorders. However, little research has focused on the sleep quality of psychiatric patients beyond depression, despite its relevance in diagnostic criteria.

**Aim:**

This study aimed to assess overall sleep quality in psychiatric outpatients and to assess for an association with socio-demographic variables.

**Setting:**

This study took place at the adult psychiatric outpatient department of Chris Hani Baragwanath Academic Hospital.

**Methods:**

A cross-sectional study design was employed to evaluate overall sleep quality using the self-administered Pittsburgh Sleep Quality Index (PSQI), a validated tool. The PSQI yields a global score ranging from 0 to 21, with scores of 5 or greater indicating poor sleep quality. Eligibility was determined through structured clinical interviews and data obtained from participant records.

**Results:**

Poor sleep quality was found in 50% of participants. Sleep quality did not differ significantly based on sex or age. Subscale analysis revealed reduced sleep duration and efficiency, nocturnal disturbances and daytime dysfunction. Additionally, 38% of participants required pharmacological intervention for sleep issues, despite lacking a diagnosis of primary or comorbid sleep disorders.

**Conclusion:**

Half of the psychiatric outpatients experienced poor sleep quality, irrespective of socio-demographic factors, psychiatric diagnosis, symptom remission or medication type.

**Contribution:**

This study highlights the importance of addressing sleep disturbances as comorbid conditions in psychiatric patients. Comprehensive evaluation and management of sleep quality can lead to improved patient outcomes and quality of life.

## Introduction

Sleep disturbance affects 50% – 80% of all patients with psychiatric disorders and can be both a cause and effect of the same.^1^ Epidemiological data from Western studies show that 0.047% – 50.5% of the general population suffer from sleep disorders.^2^ There is a downward trend in average sleep duration and an increasing prevalence of sleep disturbances.^2,3^ A significant cost burden is solely attributed to sleep problems, and this is likely to increase exponentially in the coming years. These costs are both direct (treatment costs) and indirect (absenteeism, decreased productivity, accidents, etc.) costs.^3^ Sleep disturbance is one of the features of mood, anxiety and some personality disorders, and is often bidirectional.^4,5^ Insomnia is seen in more than 90% of patients with clinical depression. Insomnia as a symptom has a 60% – 70% positive predictive value in diagnosing major depressive disorder.^2,6^ To date, most research has focussed more on sleep in depression than in any other psychiatric disorder.^7^ However, rapid eye movement (REM) sleep abnormalities have been illustrated in schizophrenia,^8,9^ alcohol use disorder, eating disorder and in borderline personality disorder.^10^

Sleep is defined as a rapidly reversible state with decreased awareness of external stimuli.^11^ It comprises several defined stages and is assessed in research by electrophysiological changes and with behavioural observation.^11^ Sleep is a universal need among species, yet the exact functions of sleep are still poorly understood. Loss of sleep, however, leads to emotional, cognitive and even physical impairment, as several sleep deprivation studies have shown.^12^

Historically, scholars in psychiatry, dating back to Sigmund Freud and Carl Jung in the 19th century, had a special interest in sleep, dreams and their relationship with mental illness.^13^ Sleep monitoring and research began as early as 1929, pioneered by Hans Berger, and by the 1970s, sleep disorders were characterised and diagnosed.^11^ Several hypotheses exist postulating the functions of sleep, but all the known facts of sleep fail to be accounted for under any one hypothesis. Broadly, current research into the functions of sleep shows a role in restoration of metabolic function and/or in serving mechanisms involved in neural plasticity.^11^

The duration of sleep obtained per night varies across the human lifespan. Natural variation of this occurs with inherently short sleepers and long sleepers. Sleep needs and patterns are influenced by several factors, including genetic factors, age, and comorbid medical and psychiatric disorders.^11^ Alteration in sleep architecture also occurs over time for example the significantly diminished amount of slow wave sleep seen during adolescence. One theory by Irwin Feinberg relates this with synaptic pruning at this stage.^11^ Abnormal synaptic pruning may account for the timing of the peak in incidence seen in schizophrenia in late adolescence and early adulthood.^14,15^ This suggests that the systems involved in sleep and wakefulness overlap significantly with emotional regulation and other behaviours.^11^ It is thus not surprising that sleep abnormalities are common in patients with psychiatric disorders, part of the diagnostic criteria of these disorders, and appear to have some predictive value for the trajectory of these disorders.^4^ Furthermore, many psychotropic medications used in the management of psychiatric disorders have both positive and negative effects on sleep.^2^

Örsal et al.^16^ defined sleep quality as ‘the efficiency of sleep’. It consists of several components: subjective sleep quality, delay to onset, duration, habitual sleep activity, primary sleep disorder, daytime dysfunction and use of opiates or sedatives. Harvey et al.^17^ proposed a two-way neurobiological relationship between sleep disturbances and emotional regulation, and how they are linked in psychiatric disorders, that is, that a functional relationship exists between sleep and mood.^17,18^ Sleep deprivation has an antidepressant-like effect, possibly by normalising the accelerated metabolic activity in the anterior cingulate gyrus seen in people with depression and anxiety disorders,^10^ which is reversed by even a short bout of sleep.

This suggests an ability for sleep loss to precipitate and perpetuate mania, and possibly other psychiatric symptoms.^17^ However, the exact causal link between health outcomes and sleep is still unclear, and controversial, and further longitudinal studies are essential.

Sleep deprivation studies in recent decades have revealed cognitive impairment, specifically, deficits in attention and short-term memory, impairment in speech, perseveration and concrete thinking.^19^ There is also an effect on emotional reactivity and processing of emotional information.^20^ Sleep deprivation may also impact on various physiological systems which may affect overall health, that is, by affecting host defences, impairing glucose tolerance, autonomic hyperarousal and increasing cortisol – which has implications for various chronic systemic disorders.^11^ These findings suggest an important correlation between sleep quality and health outcomes overall.

An association also exists between sleep disturbances and subjective benefit from treatment received, and thus, the patient’s overall perceived quality of life.^1^ Sleep disturbance also impacts objective assessment of disease severity and functioning, and has also shown to be the most common residual symptom after successful treatment of major depressive disorder.^1^

Sleep disorders and psychiatric disorders are recognised as bi-directional in the *Diagnostic and Statistical Manual of Mental Disorders, Fifth Edition* (DSM-5).^1,4,21^ Assessing sleep quality in those in remission from the core symptoms of psychiatric disorders, can help to identify the likelihood of under diagnosis of concomitant primary sleep disorders and possible treatment thereof.^1,22^ There is a paucity of evidence of sleep quality assessment in psychiatric disorders other than depression, and sleep quality directly affects the adaptation of patients to the disorder and their commitment to participating in the treatment process. This shift in thinking and greater awareness of sleep problems could assist with early recognition and management of sleep disorders, subsequently impacting on the trajectory of the disorder and remission rates, overall prognosis and quality of life.^1^ Thus, the aim of this research study was to assess the overall sleep quality of psychiatric outpatients at Chris Hani Baragwanath Academic Hospital (CHBAH), using the Pittsburgh Sleep Quality Index (PSQI), and to link this with their socio-demographic variables.

## Research methods and design

### Study design and setting

This study was a cross-sectional assessment of the overall sleep quality, and its seven components, in adult psychiatric outpatients at the CHBAH, a level 3 facility with a psychiatric department servicing southern Gauteng. The patients have a variety of psychiatric disorders (more complex cases), comorbid medical conditions, substance use disorders, require tertiary care, or cannot yet be down referred to community clinics. Several inpatients are also seen in the clinic for consultation-liaison psychiatry assessment and daily review.

### Study population and sampling strategy

The study was conducted with adult participants from the OPD at CHBAH. A clinical interview was conducted as per regular service delivery in the OPD, in conjunction with collection of data from the participants’ records (psychiatry file), to assess eligibility. Informed consent was obtained from voluntary participants who were deemed eligible for the study. A total of 90 individuals (excluding 1 repeat participant) provided informed consent and were enrolled in the study during March 2021–June 2021.

### Inclusion and exclusion criteria

The following participants were eligible for the study:

- Adults (>18 and <60 years old) with a psychiatric disorder presenting within the study period.
- Patients with residual symptoms of their disorder despite ongoing treatment.

The research study had the following exclusion criteria:

- Patients who are assessed as having comorbid substance use that is ongoing at the time of the study (by self-report and assessment for features of intoxication or withdrawal in the clinical interview) were excluded to limit confounding variables affecting sleep quality independently.
- Patients with psychiatric manifestations of another medical condition were excluded for the same reason.
- Patients requiring admission based on the clinical interview were not eligible for this study.

### Data collection and instrument (Pittsburgh Sleep Quality Index)

A clinical interview was conducted to assess current mental status, followed by cross-sectional record review to assess eligibility for the study. Data collected from eligible participant records were recorded on a data sheet and analysed. The data sheet contained the socio-demographic variables most affecting sleep quality (based on the literature review) and psychiatric diagnosis, treatment and the PSQI component and total scores. The PSQI was self-administered and collected by the principal researcher thereafter. A study number was assigned to the data sheet and corresponding PSQI to ensure confidentiality in the study.

Several methods of assessing sleep quality exist and one such tool is the PSQI, a non-specific sleep questionnaire. The PSQI was developed by Buysse et al. in 1989^23^ and has been tested and adapted (into 56 languages) for various populations since, with high validity and reliability. The PSQI is a good self-report scale used to assess subjective sleep quality, duration, latency, efficiency, disturbances, use of sleeping aids and daytime dysfunction over a 1-month period.^23^ It consists of seven subscales (component scores) scored from nine questions in the scale using the aforementioned categories and the possible score for the PSQI ranges from 0 to 21. A global score of 5 or greater is indicative of poor sleep quality.^23^ The PSQI has been validated for use in Africa^24,25^ and in South Africa,^26^ and has been used in sleep studies in South Africa,^26,27^ with specific caution suggested to be exercised with its use in shift workers.^26^ The PSQI appears to be a good rating scale for sleep quality due to its ease of use, internal consistency, proof of scores being stable over time, and its ability to identify specific cases versus control subjects (i.e. inherent good vs. poor sleepers). Self-reporting by clients, though empowering, may reflect inaccurate information for a variety of reasons including untruthful responses or incompleteness, which is a limitation of the PSQI. Permission has been granted for the PSQI to be reproduced freely in its original form, for non-commercial research and educational purposes, and requires citation only.^23^

### Data analysis

The data were captured in Microsoft Excel^TM^. All statistical analyses were conducted using R software (R version 4.0.1; https://www.r-project.org). Tests are two-tailed probability values and statistical significance is accepted when α ≤ 0.05. The data set for this study was generated from assessments of categorical (PSQI) scores. Data are presented in tables, figures and in text.

To assess the sleep quality of the sample population, the proportion of patients with scores <5 was compared to those with scores of >5 using chi-squared contingency table analyses to assess whether the proportion of patients sampled with sleep disorder was greater or less than chance.

A frequency distribution plot of the total scores was generated. The distribution of scores (from 0 to 3) for each of the seven component categories was analysed using a chi-squared contingency test.

Fisher’s exact tests were used to analyse whether socio-demographic variables (e.g. sex) predict sleep quality. For age (the continuous variable), *t*-test analysis was conducted in which age was the dependent variable and sleep quality was the independent variable.

### Ethical considerations

Permission to conduct the study was obtained from the relevant hospital authorities. Ethical clearance to conduct the study was obtained from the chairperson of the Wits Human Research Ethics Committee (Medical) – clearance number M201190. Informed consent was obtained, and participant confidentiality was maintained, with only the researchers having access to information linking the data sheet with the corresponding PSQI completed in the study. No information obtained during this study was used for purposes other than fulfilling the study objectives.

## Results

The data set included a total of 90 participants (excluding the one repeat participant whose data are not considered in the analyses) at the psychiatric OPD at CHBAH. Missing data (two incomplete questionnaires) are excluded in the analyses, below, so sample sizes varied.

### Socio-demographic data

Shift work and being the primary caregiver to children under the age of 6 years is known to affect sleep quality.^28^ Almost all patients did not do shift work (χ^2^ = 81.17, df = 1, *p* < 0.001) or provide care to young children (χ^2^ = 85.04, df = 2, *p* < 0.001). Patients in the study had a mean age of 35.2 years old (Table 1). Most patients in the study were female (57.8%) and there was one transgender patient. The distribution of the sexes did not vary significantly from chance (χ^2^ = 2.84, *df* = 1, *p* = 0.091).

**TABLE 1:** Socio-demographic variables of the study participants.

Sociodemographic variables	Number	%
**Age (years)**	35.2	10.8
**Sex**		
Female	53	58.9
Male	37	41.1
**Shift work**		
No	87	97.8
Yes	2	2.2
**Caregiver for children**		
No	88	98.9
Yes	1	1.1

### Sleep quality

**FIGURE 1:**
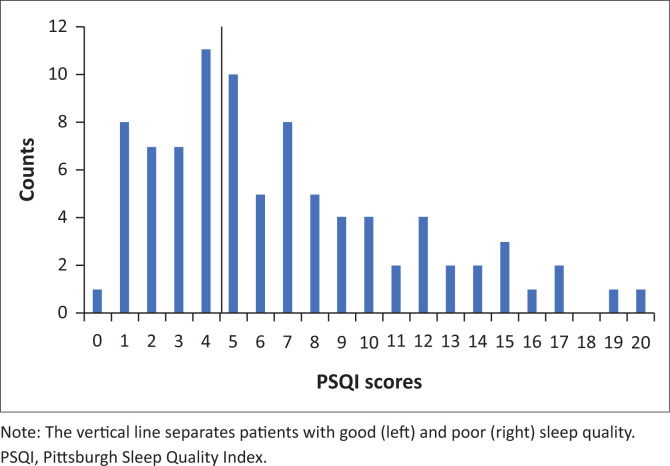
A frequency distribution of total Pittsburgh Sleep Quality Index scores in study participants.

**FIGURE 2:**
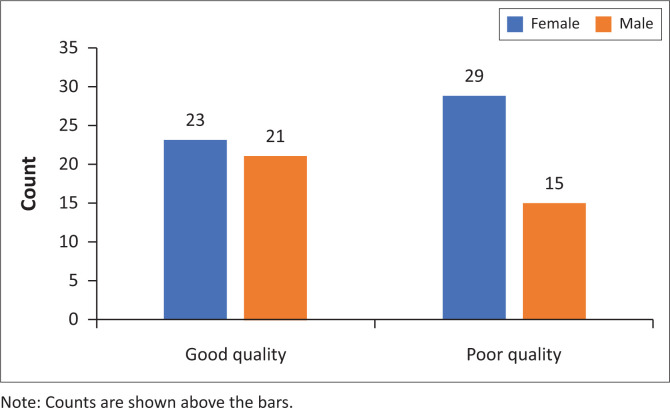
The number of female and male participants with good and poor sleep quality.

Sleep quality was categorised as good or poor, based on the total PSQI score. Sleep quality was obtained from 88 patients, with an equal number (*n* = 44) with good and poor sleep quality in the study population (Figure 2). This is not statistically significantly different from chance (χ^2^ = 0, df = 1, *p* = 1.000). A frequency plot of the total PSQI scores showed a skewed distribution (Figure 1). Because the cut off for good sleep was <5 and a similar number of the patients were categorised as having good and poor sleep quality, the left skew of the data was expected.

Table 2 shows the scores for each of seven PSQI components (subscales). The counts differed due to incomplete questionnaires being included in the subscale analysis. For the seven subscales (components) of the PSQI, the higher scores (0–3) indicated poorer sleep.

**TABLE 2:** The scores from 0 to 3 for each of the seven Pittsburgh Sleep Quality Index components and/or subscales.

PSQI score	Components (Subscales)													
Score	1 (Subjective sleep quality)		2 (Sleep latency)		3 (Sleep duration)		4 (Sleep efficiency)		5 (Sleep disturbances)		6 (Use of sleep medication)		7 (Daytime dysfunction)	
	*n*	%	*n*	%	*n*	%	*n*	%	*n*	%	*n*	%	*n*	%
0	25	28.1	27	30.7	64	72.7	64	73.6	3	3.4	55	61.8	36	40.4
1	38	42.7	20	22.7	11	12.5	16	18.4	62	69.7	5	5.6	21	23.6
2	15	16.9	15	17.0	6	6.8	1	1.1	22	24.7	4	4.5	19	21.4
3	11	12.3	26	29.6	7	8.0	6	6.9	2	2.2	25	28.1	13	14.6
*n*	89	-	88	-	88	-	87	-	89	-	89	-	89	-

Comparisons were made of the sleep quality and socio-demographic factors. The age of patients, shift work and being the primary caregiver to a young child were not significant predictors of sleep quality. Sex was not a significant predictor of sleep quality (Fisher’s exact test *p* = 0.278). Although this outcome was not significant, the proportion of sex by sleep quality showed interesting findings. For females, 44% reported good sleep quality and 56% reported poor sleep quality. In contrast, 58% of males reported good sleep quality and 42% reported poor quality (Figure 2).

Table 3 includes the diagnoses (including comorbid) of participants – which were predominantly mood disorders, psychotic disorders, personality disorders, and substance use disorders in remission. The proportion of participants with poor sleep quality was greater for mood (50.9%) than psychotic disorders (39.3%), which approached statistical significance (Fisher’s exact test = 0.061). The associated number with overall poor sleep quality as measured by the PSQI is also shown. Table 4 represents data extrapolated from the data sheet regarding sleep medications prescribed or used. Over-the-counter medications were not included in this analysis due to a lack of information regarding specific medications used. Notably, no benzodiazepines were prescribed for sleep in the study participants.

**TABLE 3:** Diagnosis and number of participants with poor sleep quality.

Diagnosis	Number of participants with diagnosis	Number with poor sleep quality (PSQI ≥5)
Schizophrenia spectrum and other psychotic disorders	33	13
Bipolar and related disorders	25	12
Depressive disorders	28	15
Anxiety disorders	7	3
Obsessive-compulsive and related disorders	1	1
Trauma- and stressor-related disorders	5	1
Personality disorders	14	10
Substance use disorders (in remission)	17	9
Other (ADHD/IDD)	3	2

ADHD, Attention-Deficit/Hyperactivity Disorder; IDD, Intellectual Developmental Disorder; PSQI, Pittsburgh Sleep Quality Index.

**TABLE 4:** Types of sleep medications and associated number of participants with the diagnoses.

Diagnostic categories	Promethazine	Quetiapine(25 mg – 150 mg)	Amitriptyline	Total
Total number of participants on this medication for sleep	15	9	2	26
Schizophrenia spectrum and other psychotic disorders	4	0	0	4
Bipolar and related disorders	5	2	0	7
Depressive disorders	4	9	2	15
Anxiety disorders	0	0	2	2
Obsessive-compulsive and related disorders	0	1	0	1
Trauma- and stressor-related disorders	1	1	0	2

## Discussion

This study was conducted at CHBAH adult OPD from March to June 2021. The number of patients serviced by the OPD lessened during the coronavirus disease 2019 (COVID-19) pandemic due to safety protocols and alteration of the number of patients booked per day. Due to the diversity of patients seen, and rigorous inclusion and exclusion criteria, only 90 patients of the total population seen at OPD were eligible for participation in the study during the study period. Because of the decrease in overall numbers seen, this led to a smaller sample size than initially anticipated.

The key findings included 50% of participants having an overall PSQI score of ≥5 and thus, poor sleep quality. This is in line with previous research placing this figure at 50% – 80% of patients with psychiatric disorders.^1^ The rigorous exclusion criteria, which excluded confounding factors such as medical conditions, active substance use and mental disorders not in remission, may account for this figure being on the lower end compared to existing research. There was no significant association between sex and age as predictors of sleep quality.

Although not significant, female participants had poorer sleep quality than male participants overall (Figure 2). Shift work (two participants) and being the primary caregiver to children under 6 years of age (one participant) were not significant predictors of sleep quality, likely due to the small data set. Although other forms of employment were not considered in this study, the presence of only two participants who did shift work could be representative of the burden of unemployment in the country (28.91%),^29^ which was worsened by job losses associated with the COVID-19 pandemic. Stigma, which still plays a role, may be an important contributing factor to the higher rates of unemployment in people living with mental illness.^30^

The diagnoses of the participants included some overlap with comorbid disorders, which are listed separately (Table 3), but broadly included: bipolar and related disorders (25 participants); depressive disorders^28^; schizophrenia spectrum and other psychotic disorders^29^; schizoaffective disorder^5^; obsessive-compulsive and related disorders^1^; trauma- and stressor-related disorders^5^; anxiety disorders^7^; substance use disorders – in remission^17^; personality disorders – cluster B^12^ and cluster C^2^; other.^4^. Mood disorders accounted for 58.9% and psychotic disorders accounted for 36.7% of the total participants, probably because mood disorders are more prevalent than psychotic disorders in the population. Although anxiety disorders are most prevalent in the general population,^31^ it is unlikely that people with this disorder alone would primarily be managed at a tertiary level institution, hence their absence from the data set.

Of the participants reporting poor overall sleep quality, 27 participants had mood disorders (50.9%), 3 had anxiety disorders that were comorbid (42.8%) and 13 had psychotic disorders (39.4%). Most previous research has focused on sleep in depression compared to other psychiatric disorders,^7^ so this finding of poor sleep predominantly in people with mood disorders is consistent with previous studies, suggesting that a functional relationship exists between sleep and psychiatric disorders, and more so, with mood disorders.^17,18^ Poor sleep quality has also been shown to impair emotional regulation.^20^

There is some value in examining the subscales rather than just the overall PSQI score.^23^ Some subscale findings are associated with emotional reactivity, emotional information processing and outcomes such as suicidality and positive affect – namely sleep duration and sleep efficiency.^20^ For example, from the results of sleep deprivation studies, sleep duration may be expected to impact emotional reactivity.^20^ Subscale 3 represents sleep duration. Thirteen participants (14.8%) had a score of 2–3 indicating sleep of less than 6 h. Sleep efficiency, which is a proxy for REM sleep and assesses time asleep vs. time in bed, was assessed on subscale 4. This showed an efficiency of <85% (score of >0) in 23 participants (26.4%). Sleep efficiency is associated with emotional information processing performance.^20^ Sleep disturbances (frequency of disruptions during the night), which are common in depression,^20^ were represented on subscale 5. Eighty-six participants (96.6%) reported sleep disturbances once or more per week. This may have an impact on daytime dysfunction (represented by subscale 7) and is associated with poorer performance in processing of emotional information.^20^ Fifty-three participants (59.6%) reported varying daytime dysfunction in activities such as staying awake while driving, eating meals, or engaging in social activities, as well as less enthusiasm in getting things done (from the PSQI). Of note, 34 participants (38%) reported use of medicines (including over-the-counter formulations) for sleep (subscale 6).

Of the participants, 26 were on medicines prescribed for sleep from the OPD, namely Promethazine (15 participants), Amitriptyline (2 participants) and Quetiapine IR 25 mg – 150 mg (9 participants), for its sedative properties at this dose.^32^ Interestingly, none of the participants enrolled in the study were prescribed or reported using benzodiazepines, and almost all the participants on sleep medicines had a mood disorder diagnosis (19 participants). However, none of these participants were diagnosed with a comorbid sleep disorder. This may have been because of the type of participants included in the study, those that were in remission from symptoms of mental illness at the time of the study and thus, have less need for these medicines. The role of education of prescribers regarding the risks of long-term benzodiazepine use and working under supervision may also be contributory.

### Strengths and limitations

The PSQI is a self-report scale, which has its own limitations as mentioned earlier. This study design was cross-sectional and only representative of the sleep quality of the sample population within the last month; further longitudinal studies are required to assess sleep quality over time.

The study did not examine PSQI subscales (only overall sleep quality) relative to diagnoses, which would have allowed for further analysis of specific sleep components and the possible association with each disorder. It also did not measure the effects of individual interventions on sleep quality or the effect of different medical comorbidities or environmental factors on poor sleep quality (e.g. body mass index, noisy residential area), but tried to limit the number of such confounding factors by the exclusion criteria.

Importantly, due to the research being conducted during periods when varying levels of restrictions on life activities were imposed nationally, the findings noted in the study may have been influenced by the effect of the COVID-19 pandemic and its accompanying psychological distress on sleep and overall well-being.^33,34,35^ Due to the relatively small sample size, these results should be interpreted with caution regarding generalisability.

### Implications

The results suggest that sleep quality and sleep disorder should possibly be conceptualised as a comorbid condition, rather than a secondary phenomenon originating from the primary psychiatric diagnosis, because participants enrolled in this study with poor sleep quality were considered to be in remission from the primary psychiatric disorder symptoms during the clinical interview, before completion of the PSQI.^1^ Treatment approaches to target remaining sleep-related symptoms include non-pharmacological interventions like sleep hygiene^36,37^ and psychotherapy (e.g. cognitive-behavioural therapy for insomnia or CBT-I)^38^; and pharmacological interventions.^1^ Extrapolating further data from the individual subscales of the PSQI, rather than the overall score, would better delineate the underlying sleep disturbance and inform management thereof.

## Conclusion

Poor sleep quality was found in half of the study participants irrespective of socio-demographic factors, medication type, psychiatric diagnosis and being in remission from symptoms. This suggests there is value in conceptualising sleep disturbances as a comorbid condition and managing it accordingly.

## References

[1] Kallestad H, Hansen B, Langsrud K, et al. Impact of sleep disturbance on patients in treatment for mental disorders. BMC Psychiatry. 2012;12:179. 10.1186/1471-244X-12-179 23107000 PMC3505143

[2] Hombali A, Seow E, Yuan Q, et al. Prevalence and correlates of sleep disorder symptoms in psychiatric disorders. Psychiatry Res. 2019;279:116–122. 10.1016/j.psychres.2018.07.009 30072039

[3] Stranges S, Tigbe W, Gómez-Olivé FX, Thorogood M, Kandala NB. Sleep problems: An emerging global epidemic? Findings from the INDEPTH WHO-SADE study among more than 40,000 older adults from 8 countries across Africa and Asia. Sleep. 2012;35(8):173–1181. 10.5665/sleep.2012 22851813 PMC3397790

[4] American Psychiatric Association1. Diagnostic and Statistical Manual of Mental Disorders. 5th ed. Arlington, VA: American Psychiatric Publishing; 2013, 947 p.

[5] American Academy of Sleep Medicine. International Classification of Sleep Disorders. 3rd ed. Darien, IL: American Academy of Sleep Medicine; 2014.

[6] Khurshid KA. Comorbid insomnia and psychiatric disorders: An update. Innov Clin Neurosci. 2018;15(3–4):28–32. PMC5906087 29707424

[7] Peterson MJ, Benca RM. Sleep in mood disorders. Psychiatr Clin N Am. 2006;29(4):1009–1032. 10.1016/j.psc.2006.09.003 17118279

[8] Benson KL. Sleep in schizophrenia. Sleep Med Clin. 2008;3(2):251–260. 10.1016/j.jsmc.2008.01.001 26055673

[9] Ferrarelli F. Sleep abnormalities in schizophrenia: State of the art and next steps. Am J Psychiatry. 2021;178(9):903–913. 10.1176/appi.ajp.2020.20070968 33726524 PMC8446088

[10] Klumpp H, Roberts J, Kapella MC, Kennedy AE, Kumar A, Phan KL. Subjective and objective sleep quality modulate emotion regulatory brain function in anxiety and depression. Depress Anxiety 2017;34(7):651–660. 10.1002/da.22622 28419607 PMC5503154

[11] Benca RM, Cirelli C, Tononi G. Basic science of sleep. In: Sadock, Benjamin J, Sadock, Virginia A, Ruiz, Pedro, editors. Comprehensive textbook of psychiatry. 9th ed. Philadelphia: Wolters Kluwer, 2009; p. 362–375.

[12] Alhola P, Polo-Kantola P. Sleep deprivation: Impact on cognitive performance. Neuropsychiatr Dis Treat. 2007;3(5):553–567. 19300585 PMC2656292

[13] Gilmore MM, Nersessian E. Freud’s model of the mind in sleep and dreaming. Neuropsychoanalysis. 1999;1(2):225–232. 10.1080/15294145.1999.10773263

[14] Feinberg I. Schizophrenia: Caused by a fault in programmed synaptic elimination during adolescence? J Psychiatr Res. 1982;17(4):319–334. 10.1016/0022-3956(82)90038-3 7187776

[15] Germann M, Brederoo SG, Sommer IEC. Abnormal synaptic pruning during adolescence underlying the development of psychotic disorders. Curr Opin Psychiatry. 2021;34(3):222–227. 10.1097/YCO.0000000000000696 33560023 PMC8048735

[16] Örsal Ö, Eren HK, Duru P. Examination of factors affecting the sleep quality of psychiatry patients using structural equation model. J Psychiatr Nurs. 2019;10(1):55–64. 10.14744/phd.2018.06978

[17] Harvey AG, Murray G, Chandler RA, Soehner A. Sleep disturbance as transdiagnostic: Consideration of neurobiological mechanisms. Clin Psychol Rev. 2011;31(2):225–235. 10.1016/j.cpr.2010.04.003 20471738 PMC2954256

[18] Winokur A. The relationship between sleep disturbances and psychiatric disorders: Introduction and overview. Psychiatry Clin N Am. 2015;38(4):603–614. 10.1016/j.psc.2015.07.001 26600099

[19] Gradinger F, Glässel A, Bentley A, Stucki A. Content comparison of 115 health status measures in sleep medicine using the International Classification of Functioning, Disability and Health (ICF) as a reference. Sleep Med Rev. 2011;15(1):33–40. 10.1016/j.smrv.2010.07.001 20817510

[20] O’Leary K, Small BJ, Panaite V, Bylsma LM, Rottenberg J. Sleep quality in healthy and mood-disordered persons predicts daily life emotional reactivity. Cogn Emot. 2017;31(3):435–443. 10.1080/02699931.2015.1126554 26756667 PMC5603277

[21] Krystal AD. Psychiatric disorders and sleep. Neurol Clin. 2012;30(4):1389–1413. 10.1016/j.ncl.2012.08.018 23099143 PMC3493205

[22] Özkan B, Çoban SA, Saraç B, Medik K. Sleep quality and factors affecting it in patients with chronic psychiatric disorders. Erciyes Med J. 2015;37(1):6–10. 10.5152/etd.2015.7837

[23] Buysse DJ, Reynolds III CF, Monk TH, Berman SR, Kupfer DJ. The Pittsburgh Sleep Quality Index: A new instrument for psychiatric practice and research. Psychiatry Res. 1989;28(2):193–213. 10.1016/0165-1781(89)90047-4 2748771

[24] Salahuddin M, Maru TT, Kumalo A, Pandi-Perumal SR, Bahammam AS, Manzar MD. Validation of the Pittsburgh Sleep Quality Index in community dwelling Ethiopian adults. Health Qual Life Outcomes. 2017;15:58. 10.1186/s12955-017-0637-5 28347341 PMC5369003

[25] Aloba OO, Adewuya AO, Ola BA, Mapayi BM. Validity of the Pittsburgh Sleep Quality Index (PSQI) among Nigerian university students. Sleep Med. 2007;8(3):266–270. 10.1016/j.sleep.2006.08.003 17368977

[26] Roche J, Vos AG, Lalla-Edward ST, Kamerman PR. Importance of testing the internal consistency and construct validity of the Pittsburgh Sleep Quality Index (PSQI) in study groups of day and night shift workers: Example of a sample of long-haul truck drivers in South Africa. Appl Ergonom. 2022;98:103557. 10.1016/j.apergo.2021.103557 34411851

[27] Iacovides S, Avidon I, Bentley A, Baker FC. Diclofenac potassium restores objective and subjective measures of sleep quality in women with primary dysmenorrhea. Sleep. 2009;32(8):1019–1026. 10.1093/sleep/32.8.1019 19725253 PMC2717192

[28] Hulsegge G, Loef B, Van Kerkhof LW, Roenneberg T, Van der Beek AJ, Proper KI. Shift work, sleep disturbances and social jetlag in healthcare workers. J Sleep Res. 2019;28(4):e12802. 10.1111/jsr.12802 30520209

[29] Chief Directorate: Advocacy and Dissemination. Statistics South Africa section 14 manual promotion of access to information [homepage on the Internet]. 2022 [cited 2023 May 15]. Available from: http://www.statssa.gov.za/wp-content/uploads/2022/09/Stats-SA-PAIA-manual-2022.pdf

[30] Gühne U, Pabst A, Löbner M, Breilmann J. Employment status and desire for work in severe mental illness: Results from an observational, cross-sectional study. Soc Psychiatry Psychiatr Epidemiol. 2021;56(9):1657–1667. 10.1007/s00127-021-02088-8 33860804 PMC8429146

[31] World Health Organization. Mental disorders [homepage on the Internet]. 2022 [cited 2023 May 17]. Available from: https://who.int.news-room/fact-sheets/detail/mental-disorders

[32] Stahl SM. Stahl’s essential psychopharmacology. 4th ed. Cambridge: Cambridge University Press; 2013, 185–187 p.

[33] Sher L. COVID-19, anxiety, sleep disturbances and suicide. Sleep Med. 2020;70:124. 10.1016/j.sleep.2020.04.019 32408252 PMC7195057

[34] Casagrande M, Favieri F, Tambelli R, Forte G. The enemy who sealed the world: Effects quarantine due to the COVID-19 on sleep quality, anxiety, and psychological distress in the Italian population. Sleep Med. 2020;75:12–20. 10.1016/j.sleep.2020.05.011 32853913 PMC7215153

[35] Duran S, Erkin Ö. Psychologic distress and sleep quality among adults in Turkey during the COVID-19 pandemic. Prog Neuropsychopharmacol Biol Psychiatry. 2021;107:110254. 10.1016/j.pnpbp.2021.110254 33485962 PMC7825837

[36] Harvey A. Sleep hygiene and sleep-onset insomnia. J Nervous Ment Dis. 2000;188(1):53–55. 10.1097/00005053-200001000-00011 10665463

[37] Rahimi A, Ahmadpanah M, Shamsaei F, et al. Effect of adjuvant sleep hygiene psychoeducation and lorazepam on depression and sleep quality in patients with major depressive disorders: Results from a randomized three-arm intervention. Neuropsychiatr Dis Treat. 2016;12:1507–1515. 10.2147/NDT.S110978 27382293 PMC4922769

[38] Smith MT, Huang MI, Manber R. Cognitive behavior therapy for chronic insomnia occurring within the context of medical and psychiatric disorders. Clin Psychol Rev. 2005;25(5):559–592. 10.1016/j.cpr.2005.04.004 15970367

